# The National Institutes of Health Stroke Scale is comparable to the ICH score in predicting outcomes in spontaneous acute intracerebral hemorrhage

**DOI:** 10.3389/fneur.2024.1401793

**Published:** 2024-07-01

**Authors:** Suzie A. Kazaryan, Kristina Shkirkova, Jeffrey L. Saver, David S. Liebeskind, Sidney Starkman, Sebina Bulic, Roy Poblete, May Kim-Tenser, Shujing Guo, Robin Conwit, Pablo Villablanca, Scott Hamilton, Nerses Sanossian

**Affiliations:** ^1^Roxanna Todd Hodges Comprehensive Stroke Program, Department of Neurology, University of Southern California, Los Angeles, CA, United States; ^2^Comprehensive Stroke Center and Department of Neurology, University of California, Los Angeles, Los Angeles, CA, United States; ^3^National Institutes of Health, Bethesda, MD, United States; ^4^Department of Neurology, Indiana University School of Medicine, Indianapolis, IN, United States; ^5^Department of Neurology, Stanford University, Stanford, CA, United States

**Keywords:** intracerebral hemorrhage, clinimetrics, prognosis, clinical trial, NIHSS

## Abstract

**Background:**

Validating the National Institutes of Health NIH Stroke Scale (NIHSS) as a tool to assess deficit severity and prognosis in patients with acute intracerebral hemorrhage would harmonize the assessment of intracerebral hemorrhage (ICH) and acute ischemic stroke (AIS) patients, enable clinical use of a readily implementable and non-imaging dependent prognostic tool, and improve monitoring of ICH care quality in administrative datasets.

**Methods:**

Among randomized trial ICH patients, the relation between NIHSS scores early after Emergency Department arrival and 3-month outcomes of dependency or death (modified Rankin Scale, mRS 3–6) and case fatality was examined. NIHSS predictive performance was compared to a current standard prognostic scale, the intracerebral hemorrhage score (ICH score).

**Results:**

Among the 384 patients, the mean age was 65 (±13), with 66% being male. The median NIHSS score was 16 (interquartile range (IQR) 9–25), the mean initial hematoma volume was 29 mL (±38), and the ICH score median was 1 (IQR 0–2). At 3 months, the mRS had a median of 4 (IQR 2–6), with dependency or death occurring in 70% and case fatality in 26%. The NIHSS and ICH scores were strongly correlated (*r* = 0.73), and each was strongly correlated with the 90-day mRS (NIHSS, *r* = 0.61; ICH score, *r* = 0.62). The NIHSS performed comparably to the ICH score in predicting both dependency or death (*c* = 0.80 vs. 0.80, *p* = 0.83) and case fatality (*c* = 0.78 vs. 0.80, *p* = 0.29). At threshold values, the NIHSS predicted dependency or death with 74.1% accuracy (NIHSS 17.5) and case fatality with 75.0% accuracy (NIHSS 18.5).

**Conclusion:**

The NIHSS forecasts 3-month functional and case fatality outcomes with accuracy comparable to the ICH Score. Widely documented in routine clinical care and administrative data, the NIHSS can serve as a valuable measure for clinical prognostication, therapy development, and case-mix risk adjustment in ICH patients.

**Clinical trial registration**Clinicaltrials.gov, NCT00059332.

## 1 Introduction

Spontaneous intracerebral hemorrhage (ICH) is associated with poor long-term outcomes and a high case fatality rate (1–3) Determining patient prognosis upon presentation is important for several reasons. It aids in counseling patients and family members, enables pretreatment severity grading to assess the efficacy of novel therapies in clinical trials, and allows for performance severity adjustment to account for case-mix differences across hospitals in quality improvement activities.

To date, the leading instrument for assessing prognosis upon presentation in ICH patients is the ICH score. This 5-item scale includes: a physical exam item - level of consciousness assessed with the Glasgow Coma Scale (GCS); a demographic item - patient age; and three neuroimaging items that include hematoma volume, presence of intraventricular hemorrhage, and infra- vs. supratentorial location of hemorrhage (4–6). The ICH score is a well-validated scale that has been shown to have substantial value in prognostication regarding patient case fatality. As a result, in the United States, the performance of an ICH Score on arrival has been endorsed by the Joint Commission and the American Heart/Stroke Association as a leading quality measure to be used in the assessment of the quality of care provided by Comprehensive Stroke Centers. In Europe and other global regions, the ICH score is sometimes used, though less widely (7, 8).

Despite its advantages, the ICH score has several drawbacks. Its assessment of patients presenting neurologic deficits is crude, confined to a 3-level classification of level of consciousness, and insensitive to deficits that are important to long-term disability, including hemiparesis, hemianopia, hemisensory loss, and aphasia. In addition to simple demographic and physical exam findings, it requires the interpretation and abstraction of neuroimaging exam findings, which can be operationally challenging. It is not available in administrative datasets, precluding its use in hospital care quality analysis by Medicare in the United States and other national and regional agencies globally. It requires acute stroke services to obtain a second prognostic measure (the ICH score) in patients in whom a first potential prognostic score [the NIH Stroke Scale (NIHSS)] has generally already been routinely obtained on arrival.

Other equally or more complex scales for ICH prognosis have been developed that incorporate additional patient features, including the history of pre-ICH cognitive impairment, the presence of hydrocephalus, the history of hypertension, admission glucose, and dialysis dependency (1–6, 9). Although many newer grading scores are predictive of outcomes, the added complexity of these scales and the lack of familiarity with them within the general medical community have limited their use.

The NIHSS offers promise as an alternative method for prognosis assessment in patients with ICH that would be simple to perform, widely available in routine clinical practice, and already incorporated into administrative datasets. The NIHSS was originally developed to assess deficit severity in patients with acute ischemic stroke. It is well-validated for that purpose and is currently the most widely used stroke severity scale for acute cerebral ischemia, with established ease of use and reproducibility in the acute setting. Whereas the ICH scale has little penetration in many countries, the NIHSS is almost universally used and is typically assessed even before ICH has been distinguished from AIS by brain imaging. It can therefore provide ultra-early prognostic stratification relevant to treatments such as blood pressure lowering that may be applied prior to brain imaging. However, the capacity of the NIHSS to provide useful prognostic information among patients with ICH has not been well-delineated.

The objective of this study was to assess the prognostic value of the NIHSS in patients with ICH and compare its prognostic performance with the ICH score.

## 2 Methods

We analyzed prospectively collected data in the Field Administration of Stroke Therapy-Magnesium (FAST-MAG) phase 3 randomized trial, which studied prehospital initiation of intravenous magnesium sulfate vs. placebo for suspected acute stroke patients presenting within 2 h from the last known well time (LKWT) (10). The study was performed at 60 receiving hospitals in Los Angeles and Orange County, California. Institutional Review Board (IRB) approval was granted at all participating sites. Explicit informed consent was obtained from patients or legally authorized representatives by off-scene enrolling physician-investigators using cellphone conversation, or patients were enrolled under an exception from informed consent regulations. The detailed methods of the FAST-MAG trial have been previously published (11, 12). Patients with suspected stroke, indicated by a positive Los Angeles Prehospital Stroke Screen (LAPSS), and within 2 h of LKWT, were enrolled in the trial.

In the trial, an early post-ED arrival study NIHSS score was performed by study coordinators, with subsequent NIHSS evaluations again performed at 24 h, 48 h, day 4, and day 90. At the same time as the early ED course NIHSS, the study nurse coordinators also performed a GCS. Functional outcome at 3 months was assessed using the modified Rankin Scale (mRS) of global disability. A central panel with access to the records of the entire hospital course and brain imaging findings adjudicated the final diagnosis of the presenting event, including acute ICH, acute intracranial hemorrhage of other types, acute cerebral ischemia, and neurovascular mimic. Regardless of the final diagnosis, all patients underwent the initial NIHSS and GCS assessments and the final 3-month mRS assessment.

Images from the first post-arrival brain imaging in each patient were sent to a central core imaging lab and read by a senior neuroradiologist. In patients with ICH, the core lab neuroradiologist assessed ICH volume using the AxBxC/2 method, the presence of intraventricular hemorrhage, and the intra- or supratentorial location. For each patient, an ICH score was calculated using the early ED course GCS score, patient age, and the Core Lab imaging findings.

All patients with a final diagnosis of spontaneous intracranial hemorrhage enrolled in the FAST-MAG trial were included in this study. The two primary outcome measures analyzed were: (1) dependency or death at 3 months, defined as a mRS of 3–6, indicating those who require some assistance with daily living, and (2) case fatality by 3 months.

Patient demographic and clinical features were characterized descriptively with means and standard deviations for parametric variables and medians and interquartile ranges (IQRs) for non-parametric variables. The relations of the NIHSS and the ICH score as continuous scores to the distribution of mRS global disability scores at 90 days were assessed using the Spearman correlation coefficient. The strength of the correlations was classified as: 0.40–0.59 – moderate; 0.6–0.79–strong; and 0.8–1–very strong (13).

For the dichotomized 90-day outcomes of dependency or death and of case fatality, the predictive performance of the full range of the NIHSS and full range of the ICH score was characterized using the c-statistic. The strength of c-statistic associations was classified as: 0.8 to 1.0– strong; 0.6 to 0.79 – moderately strong; 0.5 to 0.59 –moderate; 0.3 to 0.49 – fair; and 0 to 0.29 – poor (14). The c-statistic performance of the NIHSS and the ICH score was also quantitatively compared by testing the statistical significance of the difference between the areas under the curve (15). In addition, the NIHSS and ICH score performance at optimized cut points (Youden’s index) in forecasting the dichotomized 3-month outcomes was characterized using sensitivity, specificity, positive predictive value, and negative predictive value. The independent contribution of the NIHSS to the 90-day dependency or death outcome and the case fatality outcome were also assessed using multivariate logistic regression models, choosing variables with a univariate *p*-value of ≤0.05 as candidates.

To determine if combining the NIHSS and the ICH score yielded additional prognostic accuracy compared to each scale alone, logistic models were constructed to combine the two scales for predicting dependency or death and case fatality outcomes. The performance of the combined NIHSS and ICH score model with each scale alone was analyzed by calculating the net reclassification index (NRI), the integrated discrimination improvement (IDI), and the *p*-value for the difference in c-statistics. A *p*-value of ≤0.05 was considered statistically significant.

## 3 Results

Between January 2005 and December 2012, 384 patients with spontaneous ICH were enrolled in the FAST-MAG trial, with baseline characteristics as shown in Table 1. The mean patient age was 65 years (SD ±13) with 66% being male. The early post-arrival neurologic deficit assessments by trained study nurses were performed at a median of 148 min (IQR 121–180) after LKWT and a median of 77.9 min (49–99) after ED arrival. The NIHSS had a median of 16 (IQR 9–25) and a GCS median of 15 (IQR 10–15). Initial brain imaging was performed at a median of 89 (72–114) min after LKWT. Hematoma location was deep hemispheric in 83.1%, superficial hemispheric in 23.8%, pontine in 2.3%, cerebellar in 0.4%, and other in 3.5%, with 41.2% of cases having accompanying intraventricular hemorrhage. The mean ICH volume was 29 mL (SD 38). The ICH score was median 1 (IQR 0–2).

**Table 1:** Baseline characteristics and mRS outcomes of the intracerebral hemorrhage population.

	Overall (*N* = 384)	Good outcome (mRS 0–2) (*N* = 116)	Poor outcome (mRS 3–6) (*N* = 268)	*p*-value
Age, mean (SD), years	65 (13)	61 (12)	68 (13)	<0.001
Sex, female	33.3%	24%	37%	0.008
Race				
White	79%	78%	80%	0.58
Black	10%	13%	8%	
Asian	10%	9%	10%	
Other	2%	1%	2%	
Hispanic ethnicity	33.3%	36%	32%	0.25
Hypertension	79%	74%	81%	0.09
Diabetes	19%	14%	21%	0.08
Prestroke mRS	0 (0–0)	0 (0–0)	0 (0–0)	0.11
Time onset to paramedic on-scene, median (IQR), mins	23 (14–39)	24 (16–36)	22 (14–39)	0.36
Prehospital LAMS, median (IQR)	4 (3–5)	4 (3–5)	5 (4–5)	<0.001
Prehospital GCS, median (IQR)	15 (15–15)	15 (15–15)	15 (14–15)	<0.001
Prehospital SBP, median (IQR)	175 (25)	173 (26)	177 (25)	0.22
Prehospital DBP, median (IQR)	99 (18)	96 (180)	95 (21)	0.32
Time onset to ED arrival, median (IQR), mins	58 (45–75)	58 (46–75.5)	56 (45–75)	0.53
ED NIHSS, median (IQR)	16 (9–25)	11 (6–15)	22 (15–36)	<0.001
ED LAMS, median (IQR)	5 (4–5)	4 (3–5)	5 (5–5)	<0.001
ED GCS, median (IQR)	15 (10–15)	15 (15–15)	13 (4–15)	<0.001
Time onset to 1st imaging, median (IQR), mins	89 (74–115)	88 (74–114)	90 (74–118)	0.47
Time from 1st to 2nd imaging, median (IQR), mins	1,038 (685–1,389)	1,110 (824–1,567)	1,004 (672–1,358)	0.06
ICH Score, median (IQR)	1 (0–2)	0 (0–1)	1 (1–2)	<0.001
ICH Volume, median (IQR), mL	29 (38)	14 (18)	39 (43)	<0.001
90-day mRS, median (IQR)	4 (2–6)	2 (1–2)	5 (4–6)	<0.001

The results are reported as percentages, mean (SD), or median (IQR).mRS, modified Rankin Scale Score; LAMS, Los Angeles Motor Score; GCS, Glasgow Coma Scale Score; SBP, systolic blood pressure; DBP, diastolic blood pressure; NIHSS, NIH Stroke Scale; ICH, intracerebral hemorrhage.

**Figure 1:**
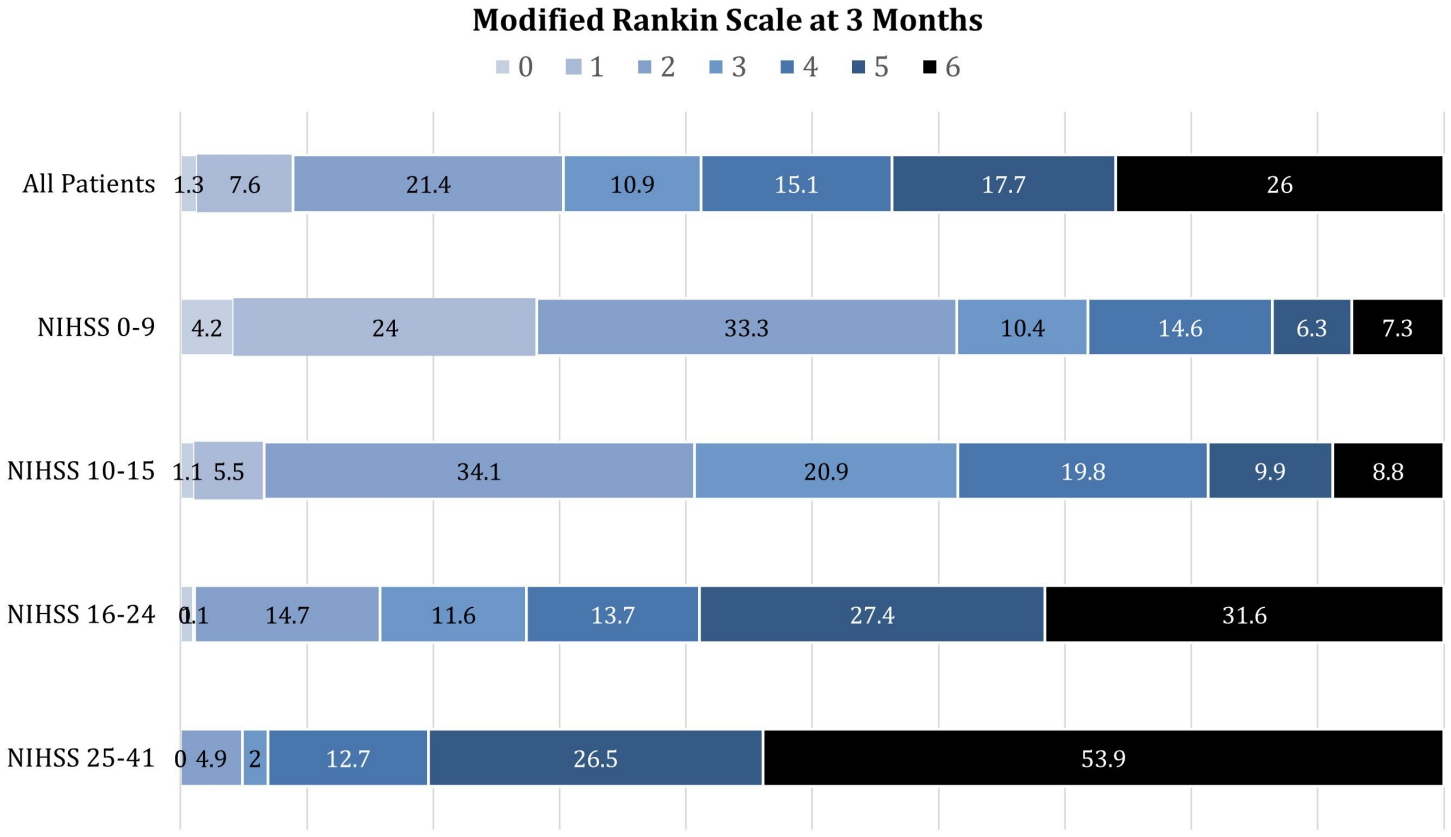
Distribution of modified Rankin Scale global disability outcomes at 3 months among patients with initial NIHSS scores of 0–9, 10–15, 16–24, and 25–41. Increasing presenting deficit severity on the NIHSS is strongly associated with the shift to worse disability outcomes at 3 months.

**Figure 2:**
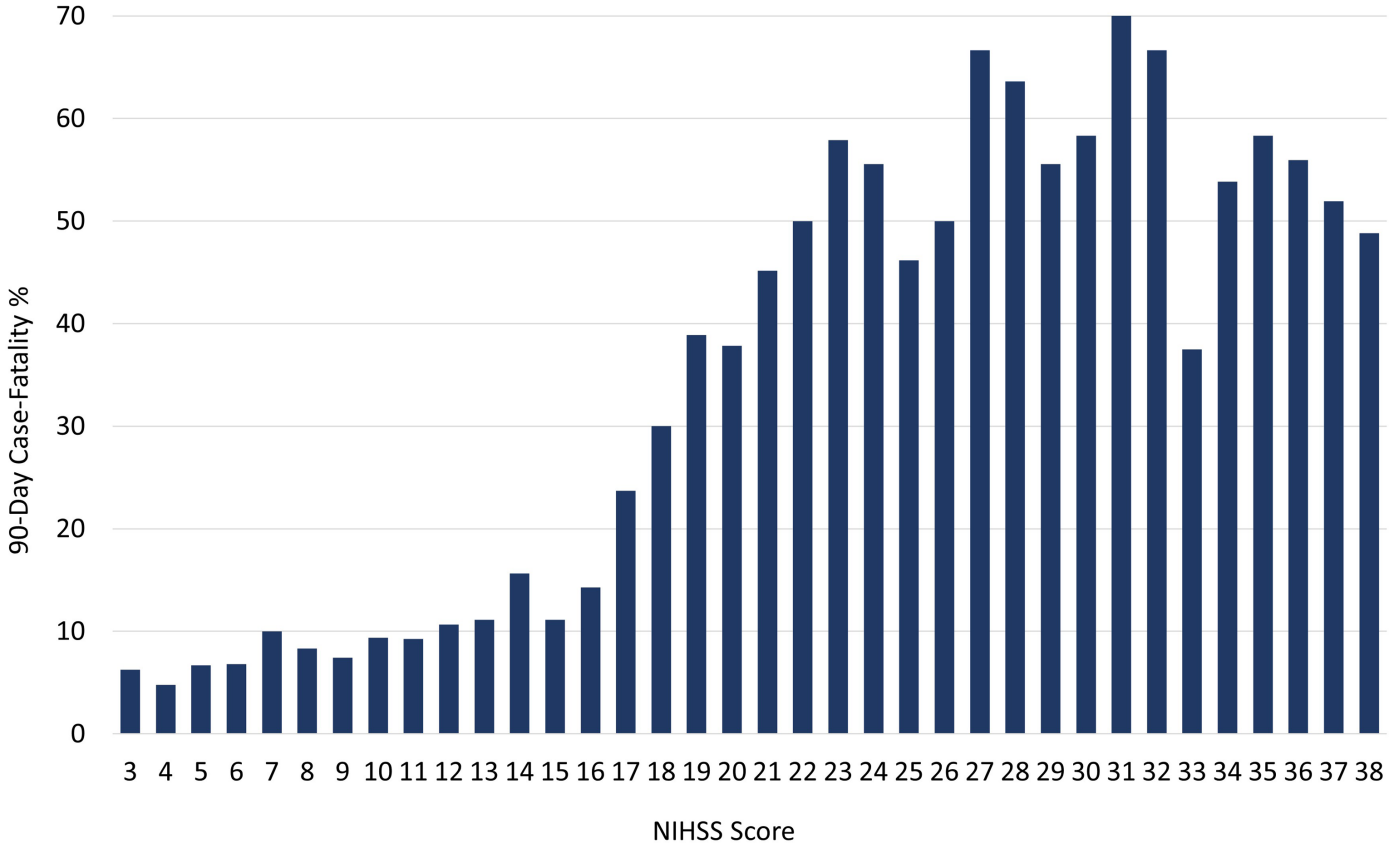
Relation of presenting NIHSS scores to 90-day case fatality rates.

The relation between presenting NIHSS scores and the distribution of mRS global disability outcomes at 90 days is shown in Figure 1. Overall, the 90-day mRS was median 4 (IQR 2–6), with a strong shift to worse outcomes with increasing presenting NIHSS severity. The relation between presenting NIHSS and 90-day case fatality is shown in Figure 2. A higher NIHSS predicted a higher case fatality risk with a c-statistic of 0.78. The case fatality rate climbed slowly from 5 to 12% as the NIHSS increased from 3 to 15, and then sharply to more than 50% as the NIHSS increased from 15 to 22.

**Figure 3:**
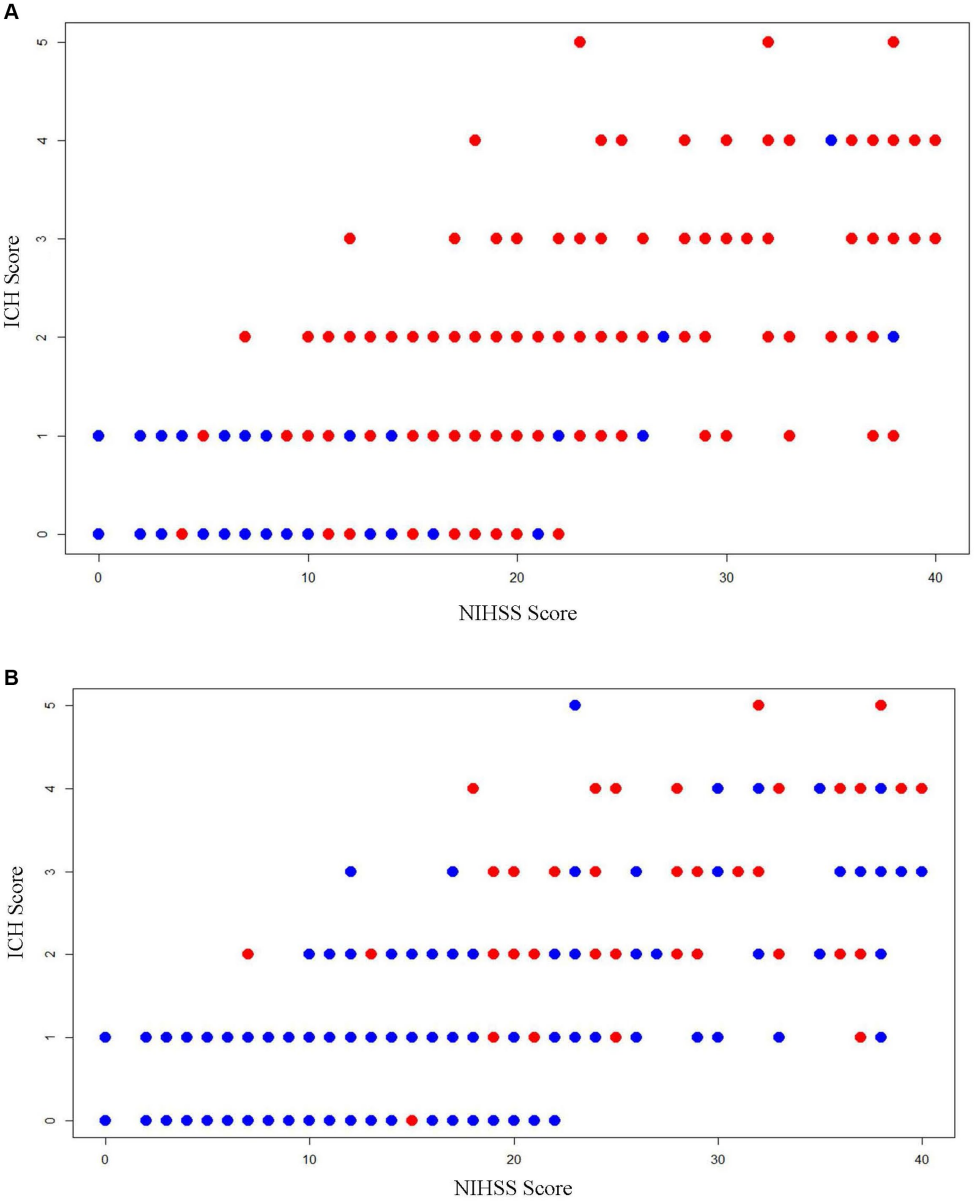
Scatterplots showing the relation of early NIHSS score, early ICH Score, and 3-month outcomes. **(A)** Dependency or death (mRS 3–6): red dots are patients with dependency or death (mRS 3–6) outcome and blue dots are patients with functional independence (mRS 0–2) outcome; **(B)** case fatality: red dots are patients with fatal outcome and blue dots are patients with the non-fatal outcome.

Strong Spearman correlations were noted between the early NIHSS and early ICH score (*r* = 0.73), early NIHSS and initial hematoma volume (*r* = 0.77), early NIHSS and 90-day mRS (*r* = 0.61), and early ICH score and 90-day mRS (*r* = 0.62) (Figure 3). In contrast, the GCS correlated less well with initial hematoma volume (*r* = 0.25) and with 90-day mRS (*r* = 0.26).

**Figure 4:**
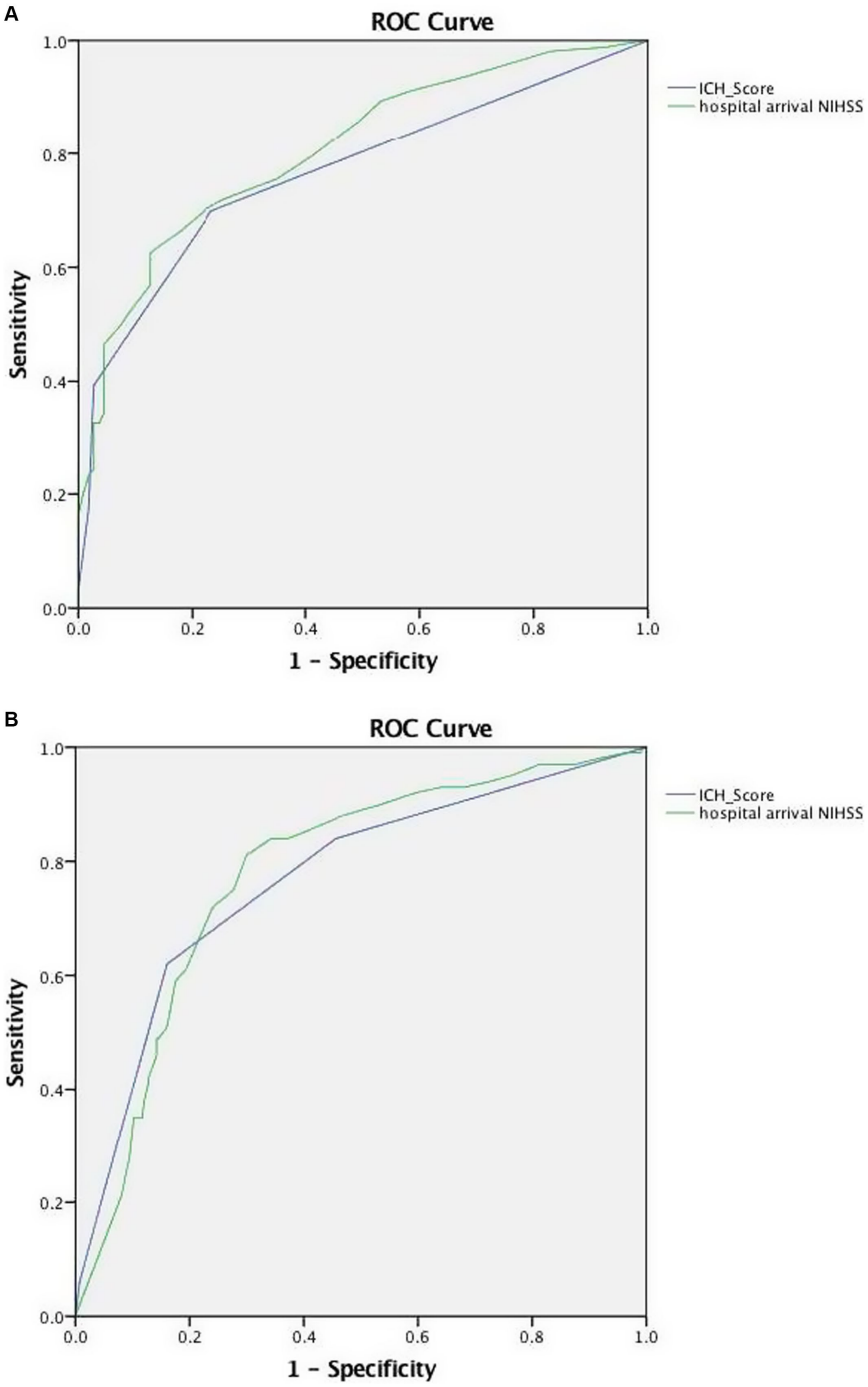
Receiver operating curves showing predictive performance of the NIHSS score and the ICH score for: **(A)** dependency or death (mRS 3–6) at 90 days and **(B)** mortality by 90 days.

The outcome of dependency or death (mRS 3–6) at 90 days occurred in 268 out of 384 (70.1%) patients, and case fatality by 90 days occurred in 100 out of 384 (26.0%) patients. The NIHSS was a strong predictor of dependency or death (mRS 3–6) at 90 days (c-statistic 0.80) and a moderately strong predictor of case fatality at 90 days (c-statistic 0.78). The ICH score performed similarly for dependency or death (mRS 3–6) at 90 days (c-statistic 0.80) and for case fatality at 90 days (c-statistic 0.80). The ROC curves showed similar performance NIHSS and ICH scores for predicting dependency or death (*p* = 0.83) and case fatality (*p* = 0.29) (Figure 4). In contrast, the GCS alone performed somewhat less well, though still moderately strongly, in predicting dependency or death (c-statistic 0.71) and case fatality (c-statistic 0.73). At optimized cutpoints, both the NIHSS alone and the ICH score alone showed good to excellent specificity, sensitivity, PPV, NPV, and overall accuracy in forecasting the 3-month outcomes (Table 2).

**Table 2:** Predictive performance of NIHSS and ICH score for 90-day outcomes.

	NIHSS	ICH score	NIHSS+ICH score combined
Dependency or death (mRS 3–6) 90 days			
C-statistic	0.80	0.80	0.84*
Performance at optimal cutpoint			
Cutpoint value	17.5	1.5	0.825
Sensitivity	61.6%	51.2%	69.0%
Specificity	86.6%	95.8%	87.8%
Positive predictive value	91.5%	96.6%	93.0%
Negative predictive value	49.0%	33.8%	54.7%
Overall accuracy	74.1%	73.5%	78.4%
Case fatality by 90 days			
C-statistic	0.81	0.80	0.81**
Performance at optimal cutpoint			
Cutpoint value	18.5	1.5	−0.899
Sensitivity	80.0%	76.0%	73.0%
Specificity	70.0%	77.0%	82.2%
Positive predictive value	48.4%	53.7%	59.0%
Negative predictive value	90.9%	90.1%	89.7%
Overall accuracy	75.0%	76.5%	77.6%

^*^Logit model = −1.17427 + 0.90916 ICH score + 0.07519 NIHSS.

^**^Logit model = −2.74657 + 0.68034 ICH score + 0.02631 NIHSS.

Combining the NIHSS and the ICH score in a single prognostic model yielded mildly further improved predictive power for dependency or death (c-statistic 0.84), with sensitivity particularly improved at an optimized cutpoint (Table 2). Combining the NIHSS and the ICH score did not yield an overall major change in predictive power for case fatality (c-statistic 0.81), but specificity was improved. Assessment of the incremental value of combining the NIHSS and the ICH score vs. using either scale alone showed a modest gain. For dependency or death, the combined model vs. the ICH score showed an NRI of 0.283, an IDI of 3.87%, and a c-statistic comparison *p*-value of 0.002. The combined model vs. the NIHSS showed an NRI of 0.077, an IDI of 5.21%, and a c-statistic comparison *p*-value of 0.01. For case fatality, the combined model vs. the ICH score showed an NRI of 0.122, an IDI of 0.32%, and a c-statistic comparison *p*-value of 0.10. The combined model vs. the NIHSS showed an NRI of 0.007, an IDI of 6.42%, and a c-statistic comparison *p*-value of 0.02. On multivariate analysis, age (OR 1.07, 95%CI 1.04–2.0) and an increase in ED NIHSS by one point (OR 1.18, 95%CI 1.13–1.23) were independent predictors of poor outcome.

## 4 Discussion

In this multicenter study involving patients with acute ICH, the NIHSS was assessed at a median of 2 h and 28 min after onset. The level of disability, presence of dependency, and case fatality outcomes at 3 months were predicted with high accuracy, comparable to the performance of the ICH Score, which is considered a current gold standard prognostic instrument. The early NIHSS showed a strong correlation with the full range of global disability outcomes at 3 months across all seven levels of the mRS. For the dichotomized outcome of dependency or death at 3 months, the NIHSS showed strong sensitivity and very strong specificity. For case fatality, the NIHSS showed very strong sensitivity and strong specificity. Combining the NIHSS and the ICH score in unified models mildly improved the prognostic performance of each score alone.

The robust prognostic performance of the NIHSS for ICH parallels its performance for acute ischemic stroke and accords with its design as a comprehensive clinical score that assesses multiple neurologic domains, including the level of consciousness, language, motor and sensory function, and coordination (16). As the NIHSS maps multiple functional domains, not just the level of consciousness, it predicts functional outcomes better than the more focused GCS alone. Furthermore, the NIHSS’ sampling of multiple brain region functions also results in its correlating closely with hematoma volumes. As a result, the NIHSS reflects, in a single assessment, the distinctive information carried in the separate GCS clinical score and in hematoma volume radiologic measurement, which are combined in the ICH Score.

The findings of this study are in agreement with and, importantly, extend prior investigations. Several prior studies have shown that admission NIHSS correlates with an in-hospital, 30-day, or 90-day case fatality, but did not investigate long-term functional outcomes nor compare performance with the ICH Score (17–19). A prior study of the detailed relation between admission NIHSS and admission hematoma volume in the FAST-MAG dataset demonstrated a close correlation (20). A single-center study showed a correlation between admission NIHSS and functional independence at 3 months but did not compare predictive performance to the ICH score (21). That study showed an even stronger correlation than the current investigation between the admission NIHSS and 3-month functional outcomes, likely because it included patients arriving later than the hyperacute FAST-MAG population who were still in a period of dynamic change in hematoma volume when first assessed (22, 23).

It is important to note that the GCS performed moderately well in forecasting the dichotomous outcomes of death or dependency (mRS 3–6) and case fatality (mRS 6) at 90 days. As GCS is performed widely and does not require extensive training, this success in predicting dichotomous outcomes indicates it will remain a useful parameter at sites without additional training resources. However, the GCS performed poorly in discriminating among all seven mRS levels of disability concurrently, whereas the NIHSS performed well.

The relation of presenting NIHSS to case fatality in ICH patients in the current study contrasts with that among ischemic stroke patients in a prior Get with the Guidelines–Stroke study. Among ICH patients, the case fatality rate climbed slowly from 5 to 12% as the NIHSS increased from 3 to 15, then sharply to more than 50% as the NIHSS increased from 15 to 22, followed by a plateau. In contrast to this S-shaped curve, among ischemic stroke patients’ cases, fatality rates increased linearly as the NIHSS increased from 1 to 35 (16). In part, this may reflect that ICHs are resorbed over time so that case fatality is dependent upon an ICH volume threshold sufficient to cause herniation or other acute complications, while ischemic stroke lesion size persists over time, exerting an ongoing effect toward case fatality.

Given that the NIHSS performs comparably to the ICH Score, several pragmatic advantages support the use of the NIHSS as a severity and prognostic measure. First, it is already nearly universally obtained and documented in clinical practice in ICH patients, as first NIHSS assessments are typically performed in newly arriving acute stroke patients in the emergency department before brain imaging has segregated patients into ischemic or hemorrhagic bins. Second, personnel trained to perform the NIHSS are widely available among emergency department nursing staff, emergency physicians, and acute stroke teams, whereas ICH score expertise is less widespread. Third, the NIHSS may be scored by a rater based on a single episode of interaction with the patient, while the ICH score requires integrating information from separate clinical and radiology finding sources. Fourth, the NIHSS codes are included in the International Classification of Diseases administrative data system, making them readily available for risk-adjusted analyses of care quality by health system planners (24).

The current study has several strengths, including being performed at multiple hospitals in an ethnically diverse geographic region with clinical trial-level monitoring of data quality. The study also has limitations. First, it compared the NIHSS with the ICH score but not with other available prognostic scales and models for ICH, of which there are more than 50 (25). However, a recent meta-analysis found that the ICH score is comparable or better in performance and most extensively validated of existing scales, making it most suitable for comparison (26). Second, the FAST-MAG trial population may not be fully representative of the ICH population as a whole. Although the trial had broad entry criteria, patients with extremely elevated blood pressure (initial systolic blood pressure > 220 mmHg), very mild (absence of any motor deficit or rapidly improving symptoms), or very severe deficits (coma in the field), or age less than 40 were excluded. Third, this was a retrospective analysis of already collected data, not a prospective cohort study. Fourth, the community hospital work-up was insufficient to reliably characterize the frequency of different hemorrhage etiologies, such as hypertensive arteriopathy or amyloid angiopathy. Fourth, the trial enrolled only patients presenting to prehospital care within 2 h of the last known well. The course in later presenting patients may differ somewhat. Furthermore, the study population was of moderate size. Accordingly, study results require replication in additional, broad patient samples.

In conclusion, the NIHSS predicts 3-month disability and case fatality outcomes with substantial accuracy, performing comparably with the ICH score. Widely documented in routine clinical care and administrative datasets, the NIHSS deficit severity score can serve as a valuable measure for clinical prognostication, therapy development, and case-mix risk adjustment in patients with ICH.

## Data availability statement

Publicly available datasets were analyzed in this study. This data can be found here: https://www.ninds.nih.gov/current-research/research-funded-ninds/clinical-research/archived-clinical-research-datasets.

## Ethics statement

The studies involving humans were approved by UCLA Institutional Review Board. The studies were conducted in accordance with the local legislation and institutional requirements. The participants provided their written informed consent to participate in this study.

## Author contributions

SK: Writing – original draft. KS: Writing – original draft. JS: Writing – original draft. DL: Writing – review & editing. SS: Writing – review & editing. SB: Writing – review & editing. RP: Writing – review & editing. MK-T: Writing – review & editing. SG: Writing – review & editing. RC: Writing – review & editing. PV: Writing – review & editing. SH: Writing – review & editing. NS: Writing – review & editing.
